# Stress-induced phosphoprotein 1 acts as a scaffold protein for glycogen synthase kinase-3 beta-mediated phosphorylation of lysine-specific demethylase 1

**DOI:** 10.1038/s41389-018-0040-z

**Published:** 2018-03-29

**Authors:** Chia-Lung Tsai, An-Shine Chao, Shih-Ming Jung, Chiao-Yun Lin, Angel Chao, Tzu-Hao Wang

**Affiliations:** 1 Genomic Medicine Research Core Laboratory, Chang Gung Memorial Hospital, Taoyuan, Taiwan; 2 Department of Obstetrics and Gynecology, Chang Gung Memorial Hospital, Linkou Medical Center and Chang Gung University, Taoyuan, Taiwan; 3 Department of Pathology, Chang Gung Memorial Hospital, Linkou Medical Center and Chang Gung University, Taoyuan, Taiwan; 4 Gynecologic Cancer Research Center, Chang Gung Memorial Hospital, Taoyuan, Taiwan

## Abstract

Stress-induced phosphoprotein 1 (STIP1)—a co-chaperone of heat shock proteins—promotes cell proliferation and may act as an oncogenic factor. Similarly, glycogen synthase kinase-3 beta (GSK3β)-mediated phosphorylation of lysine-specific demethylase 1 (LSD1)—an epigenetic regulator—can contribute to the development of an aggressive cell phenotype. Owing to their ability to tether different molecules into functional complexes, scaffold proteins have a key role in the regulation of different signaling pathways in tumorigenesis. Here, we show that STIP1 acts as a scaffold promoting the interaction between LSD1 and GSK3β. Specifically, the TPR1 and TPR2B domains of STIP1 are capable of binding with the AOL domain of LSD1, whereas the TPR2A and TPR2B domains of STIP1 interact with the kinase domain of GSK3β. We also demonstrate that STIP1 is required for GSK3β-mediated LSD1 phosphorylation, which promoted LSD1 stability and enhanced cell proliferation. After transfection of cancer cells with double-mutant (S707A/S711A) LSD1, subcellular localization analysis revealed that LSD1 was translocated from the nucleus to the cytoplasm. In vitro experiments also showed that the LSD1 inhibitor SP2509 and the GSK3β inhibitor LY2090314 acted synergistically to induce cancer cell death. Finally, the immunohistochemical expression of STIP1 and LSD1 showed a positively correlation in human cancer specimens. In summary, our data provide mechanistic insights into the role of STIP1 in human tumorigenesis by showing that it serves as a scaffold for GSK3β-mediated LSD1 phosphorylation. The combination of LSD1 and GSK3β inhibitors may exert synergistic antitumor effects and deserves further scrutiny in preclinical studies.

## Introduction

Stress-induced phosphoprotein 1 (STIP1, also known as heat shock protein [HSP] 70/90 organizing protein, Gene ID 10963) is a 62.6-kDa protein that acts as a co-chaperone of HSPs. It is structurally characterized by the presence of three tetratricopeptide repeat (TPR) domains as well as two domains rich in aspartate and proline (DP domains)^1,2^. Within the HSP90 chaperone machinery, the TPR and DP2 domains are capable of interacting with the HSP90 and HSP70 proteins^3–5^. Knockout mice lacking STIP1 are embryonic lethal, suggesting a key developmental role for this molecule^6^. Growing evidence also indicates that STIP1 is markedly overexpressed in various human solid malignancies^7–13^. Conversely, its repression blocks both tumor cell proliferation^11^ and migration^14^. At the molecular level, the anticancer effect of STIP1 blockade is accompanied by a decreased expression of HSP90 client proteins^14^ as well as inhibition of the JAK2-STAT3 pathway^5^.

Histone lysine-specific demethylase 1 (LSD1; also known as KDM1A, Gene ID 23028)—a major epigenetic regulator—is capable of removing methyl groups from histone H3 lysine 4 (H3K4) or histone H3 lysine 9 (H3K9)^15^. LSD1 is structurally characterized by the presence of three major domains, i.e., an N-terminal SWIRM domain, a central protruding tower domain, and a C-terminal amine oxidase like (AOL) domain^16^. Besides catalyzing histone demethylation, LSD1 is capable of interacting with other proteins involved in oncogenesis (including DNMT1 and p53)^17^. Importantly, it also acts as a prosurvival factor^18^ and is overexpressed in numerous cancers^19–22^. Several kinases are able to regulate the biological function of LSD1 through phosphorylation^11,23^. For example, protein kinase Cα (PKCα)-mediated LSD1 phosphorylation at serine 112 activates gene expression^24^ and promotes the acquisition of a metastatic phenotype in breast cancer^23^. Moreover, casein kinase 2 (CK2)-mediated LSD1 phosphorylation at serine 131 and serine 137 activates the DNA repair machinery^25^ and could serve as a target for the development of anticancer drugs.

Glycogen synthase kinase-3 beta (GSK3β)—a serine/threonine kinase involved in the regulation of multiple signaling pathways—recognizes substrates containing a short consensus phosphorylation (S/T)XXX(S/T) motif^26,27^. Although knockdown of GSK3β has been shown to suppress tumor cell growth and proliferation in some studies^28,29^, this effect is variable and might be context-dependent^30,31^. This phenomenon may at least in part be explained by ability of GSK3β to interact with different specific substrates.

Owing to their ability to tether different molecules into functional complexes, scaffold proteins have a key role in the regulation of different signaling pathways in tumorigenesis^32^. In this regard, HSP90 is capable of forming complexes with LSD1 to regulate estrogen receptor-mediated transcription^22^ and may bind with both β-catenin and GSK3β. Because the GSK3β-mediated β-catenin phosphorylation is blocked by HSP90 inhibitors^33^, we reasoned that the STIP1–HSP90 complex could interact with LSD1 and GSK3β to regulate LSD1 function in human oncogenesis. In the current study, we demonstrate that the STIP1–HSP90 complex is involved in GSK3β-mediated LSD1 phosphorylation by acting as scaffold that transfers LSD1 to GSK3β. Our data ultimately provide novel mechanistic insights into the role of STIP1 in tumorigenesis.

## Results

### STIP1 is capable of interacting with both LSD1 and GSK3β to form complexes

**Fig. 1:**
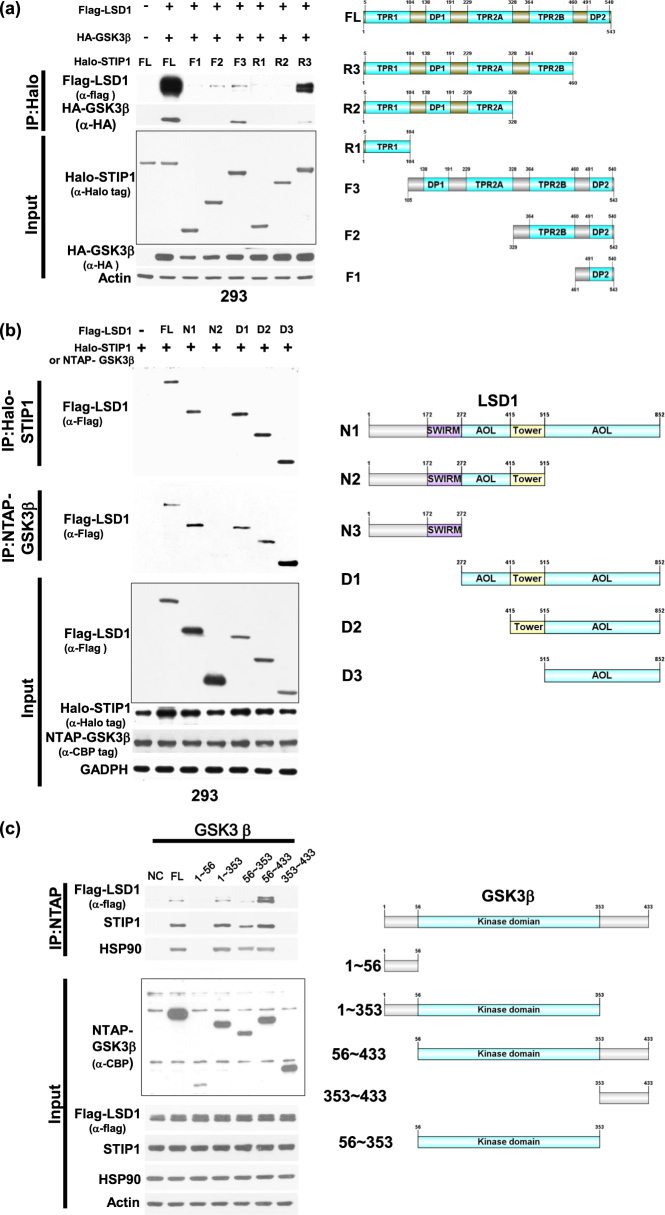

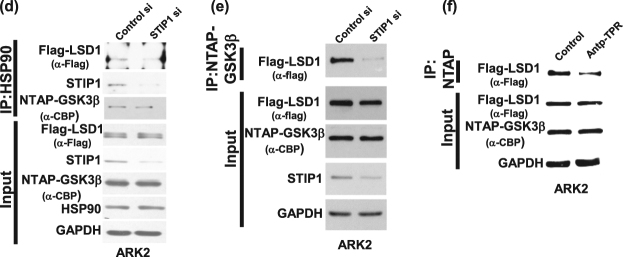
STIP1 is required for the formation of complexes containing LSD1 and GSK3β. **a** As far as STIP1 is concerned, we used the following Halo-tagged constructs: (1) the full-length (FL) protein, (2) N-terminal deleted halo-tag STIP1 constructs (F3: TPR1 deleted, F2: TPR1-DP1-TPR2A deleted and F1: TPR1-DP1-TPR2A-TPR2B deleted), and (3) C-terminal deleted halo-tag STIP1 constructs (R3: DP2 deleted, R2: TPR2B-DP2 deleted, and R1: DP1-TPR2A-TPR2B-DP2 deleted). The constructs were co-transfected with Flag–LSD1 or HA-GSK3β in 293 cells and subsequently purified with Halo-tag resin. The interactions between LSD1 and GSK3β were examined with Western blot. **b**, **c** 293 cells were co-transfected with (1) FL flag–LSD1, (2) its deleted N-terminal Flag–LSD1 constructs (D1: amino acid 272−852, D2: amino acid 415−852, and D3: amino acid 515−852), (3) its deleted C-terminal Flag–LSD1 constructs (N1: amino acid 1−515, D2: amino acid 1−272), and (4) FL Halo-STIP1 or NTAP–GSK3β. After purification with a halo-tag resin (Halo-STIP1) or streptavidin beads (NTAP–GSK3β), co-immunoprecipitated Flag–LSD1 constructs were analyzed with Western blot using an anti-flag tag antibody. **c** The identification of GSK3β domains involved in the interaction was performed by immunoprecipitation using a streptavidin resin to pull-down NTAP–GSK3β constructs. Full-length NTAP–GSK3β or its truncated constructs—including deleted N-terminal NTAP–GSK3β constructs (amino acid 56−433 and 353−433), deleted C-terminal NTAP- GSK3β constructs (amino acid 1−56 and 1−353) and a kinase domain construct (amino acid 56−353) were co-transfected with full-length Flag–LSD1 in 293 cells. Co-immunoprecipited HSP90, STIP1, and Flag–LSD1 were analyzed with Western blot using anti-HSP90, anti-STIP1 and anti-Flag antibodies, respectively. NTAP–GSK3β constructs were detected with an anti-calmodulin binding peptide (CBP) antibody. **d** The endogenous HSP90 complex in ARK2 cells were immunoprecipitated with an anti-HSP90 antibody in presence of scrambled siRNA or STIP1 siRNA. The proteins interacting with HSP90 (i.e., STIP1, LSD1, and GSK3β) were identified with Western blot. **e**, **f** The interaction between Flag–LSD1 and NTAP–GSK3β in ARK2 cells was assayed by precipitation with streptavidin beads in presence of scrambled siRNA, STIP1 siRNA (**e**), or vehicle control (PBS) and Antp-TPR (20 µM) (**f**). The STIP1, NTAP–GSK3β, and Flag–LSD1 complexes were analyzed with Western blot

To investigate whether STIP1 was capable of interacting with both LSD1 and GSK3β in living cells, systematically truncated constructs of STIP1^5^ were used to pull-down complexes. The deletion of TPR1 in F3/STIP1 and the deletion of TPR2B in R2/STIP1 (Fig. 1a) markedly decreased the capacity of STIP1 to bind LSD1. Moreover, the deletion of TPR2A in F2/STIP1 and the deletion of TPR2B in R2/STIP1 resulted in a diminished STIP1/GSK3β interaction. Interestingly, deletion of the AOL domains in N2/LSD1 reduced both the LSD1/STIP1 and the LSD1/GSK3β interactions (Fig. 1b). Conversely, the C-terminal segment of AOL domain (D3/LSD1) in LSD1 was sufficient to allow binding to both STIP1 and GSK3β (Fig. 1b). Notably, the kinase domain of GSK3β (from amino acid 56 to amino acid 353) was required to ensure the interaction with both STIP1 and LSD1 (Fig. 1c). Taken together, these results suggest that STIP1 can form complexes with both LSD1 and GSK3β. Specifically, the TPR1 and TPR2B domains of STIP1 are essential for mediating its interaction with LSD1, whereas its TPR2A and TPR2B domains are necessary for its binding to GSK3β. The C-terminal AOL domain of LSD1 mediates the interaction of STIP1 with GSK3β. Finally, the kinase domain of GSK3β is critical for interaction between STIP1 and LSD1. Of note, in all of these experiments, we cannot exclude the role of endogenous HSP90.

### STIP1 tethers both LSD1 and GSK3β

Knockdown of endogenous STIP1 inhibited the binding of LSD1 to both HSP90 (Fig. 1d) and GSK3β (Fig. 1e), although it did not affect the HSP90/GSK3β interaction (Fig. 1d). Antp-TPR—a peptide derived from the TPR2A domain of STIP1—is capable of blocking the formation of the STIP1–HSP90 complex^5,34^. To investigate the role played by STIP1–HSP90 in mediating the interaction between LSD1 and GSK3β, cancer cells were treated with the Antp-TPR peptide^34^. The results showed that 20 μM Antp-TPR peptide partially blocked the LSD1/GSK3β interaction (Fig. 1f), suggesting a role for the STIP1–HSP90 complex in tethering both LSD1 and GSK3β.

### LSD1 is a substrate for GSK3β-mediated phosphorylation

**Fig. 2:**
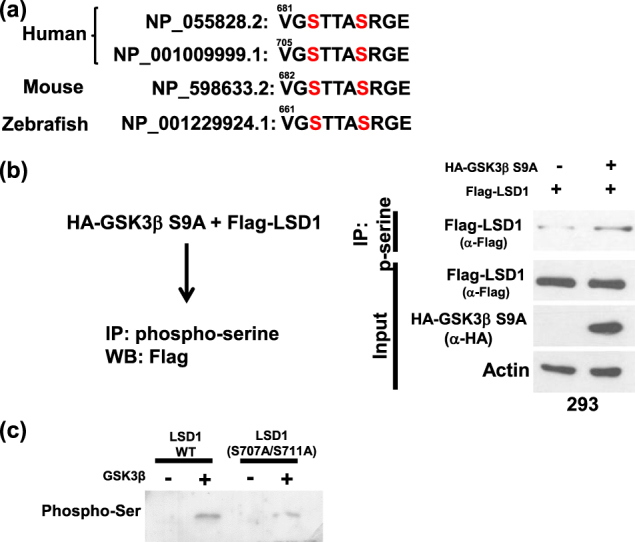
LSD1 is a substrate of GSK3β. **a** GSK3β phosphorylation motif in two human LSD1 isoforms and in the mouse and zebrafish LSD1. **b** After co-transfection of constitutively activated HA-GSK3β S9A and Flag–LSD1 into 293 cells, the phospho-serine proteins were pulled down with an anti-phospho-serine antibody, whereas Flag–LSD1 was identified with Western blot. **c** In vitro kinase assay. Recombinant Halo-LSD1 667−852 wild-type and Halo-667–852 double-mutant (S707A/S711A) LSD1 were overexpressed in 293 cells and purified with a halo resin. After incubation with GSK3β in presence of ATP, phosphorylated LSD1 was identified with an anti-phospho-serine antibody

Figure 2a shows the location of the predicted GSK3β phosphorylation motif (STTAS) along various LSD1 protein sequences. Pull-down experiments (performed with anti-phospho-serine) on lysates from cells co-transfected with LSD1 and constitutively active GSK3β (HA-GSK3β S9A) showed increased phospho-LSD1 protein levels (Fig. 2b). In an in vitro kinase assay, wild-type or double-mutant (S707A/S711A, which is unable to undergo effective GSK3β-mediated phosphorylation) LSD1 were subsequently purified and incubated with GSK3β in presence of adenosine triphosphate (ATP). The results indicated that the double-mutant (S707A/S711A) LSD1 resulted in lower phospho-serine levels compared with wild-type LSD1 (Fig. 2c), suggesting that wild-type LSD1 can effectively undergo GSK3β-mediated phosphorylation.

### GSK3β-mediated LSD1 phosphorylation promotes LSD1 protein stability

**Fig. 3:**
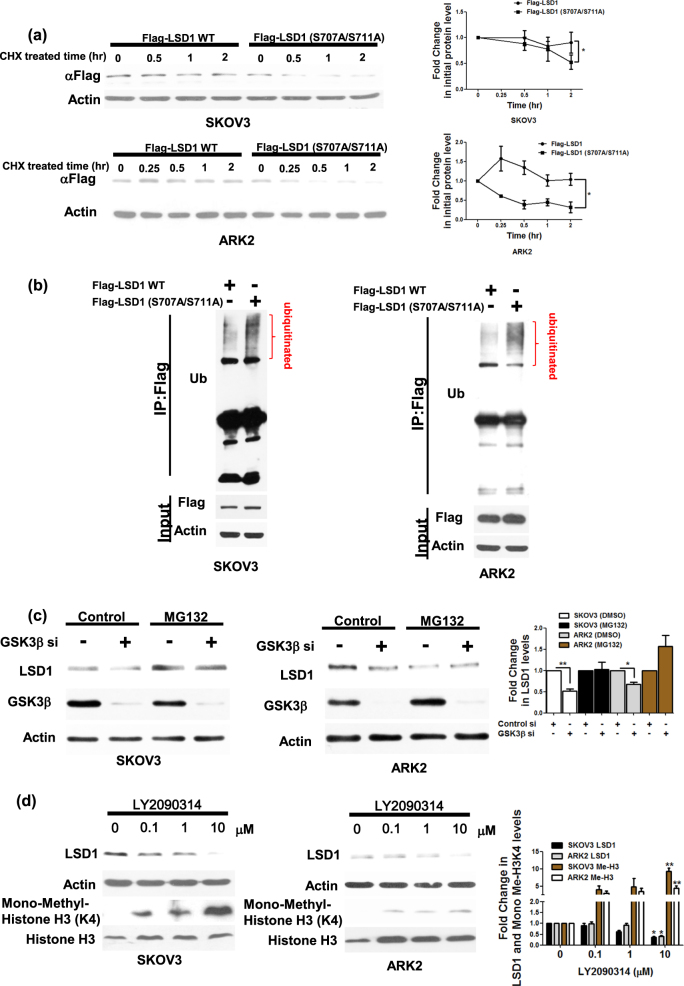

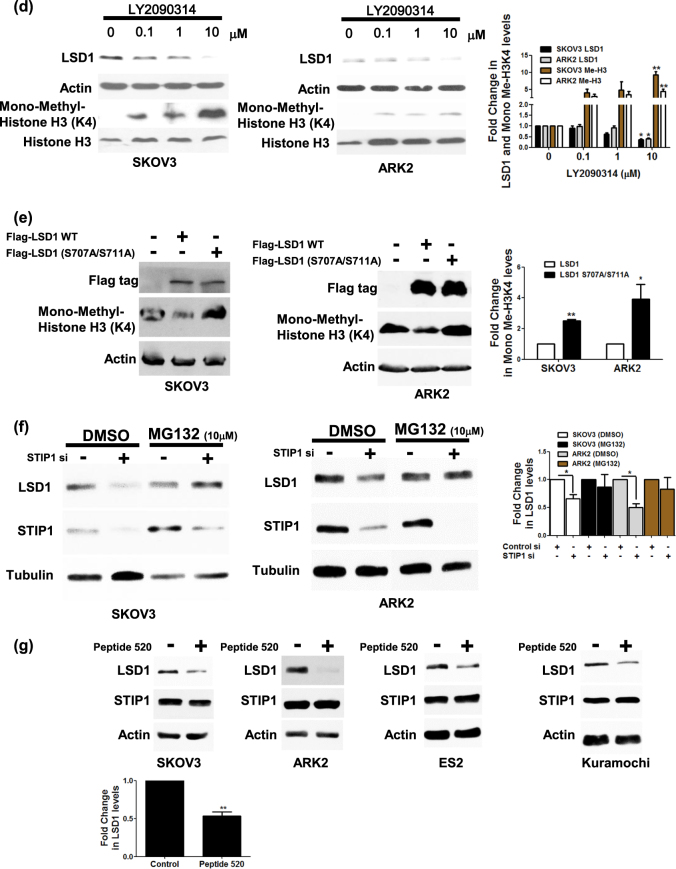
GSK3β maintains LSD1 stability. **a** Ovarian (SKOV3) and endometrial (ARK2) cancer cells were transfected with Flag-wild-type LSD1 or Flag-double-mutant (S707A/S711A) LSD1. Overexpressed LSD1 protein levels were determined by Western blot with an anti-Flag antibody in presence of 25 µg/ml cycloheximide (CHX) at the indicated time point. Actin levels were used to normalize the input proteins. **b** After treatment with 25 µM MG132 for 6 h, exogenous Flag-wild-type LSD1 or Flag-double-mutant (S707A/S711A) LSD1 in cancer cells (SKOV3 and ARK2) were immunoprecipitated with an anti-Flag antibody. Ubiquitin-conjugated, Flag-wild-type LSD1, or Flag-double-mutant (S707A/S711A) LSD1 were identified with Western blot using an anti-ubiquitin antibody. Protein lysates (50 µg) were used as a loading control and probed with anti-Flag and anti-actin antibodies. **c** Cancer cells (SKOV3 and ARK2) were treated with MG132 (25 µM) for 6 h either with or without GSK3β siRNA. Endogenous LSD1 and GSK3β protein levels were determined with Western blot. **d** SKOV3 and ARK2 cells were treated for 24 h with different concentrations of the GSK3β inhibitor LY2090314. Protein levels of LSD1, mono-methylated H3K4, total histone 3, and actin were determined with Western blot. **e** SKOV3 and ARK2 cells were transfected with Flag-wild-type LSD1 or Flag-double-mutant (S707A/S711A) LSD1. Protein levels of Flag-wild-type LSD1 or Flag-double-mutant (S707A/S711A) LSD1, mono-methylated histone H3K4, and actin were determined with Western blot. **f** After STIP1 siRNA transfection for 72 h, SKOV3 and ARK2 cells were treated with MG132 (25 µM) for 6 h. Untreated cells served as controls. Protein levels of LSD1, STIP1, and tubulin were determined with Western blot. **g** Cancer cells (SKOV3, ARK2, ES2, and Kuramochi) were treated with peptide 520 (10 µM) for 72 h in serum-free medium. Endogenous protein levels of LSD1, STIP1, and actin were determined with Western blot. Results shown were obtained from three independent experiments and are presented as mean ± standard error (SE). *P < 0.05 and **P < 0.01, Student’s *t*-test

To investigate LSD1 function in relation to GSK3β-mediated phosphorylation, we compared the protein stability of wild-type LSD1 versus double-mutant (S707A/S711A) LSD1 when overexpressed in cancer cells in presence of cycloheximide. Protein levels of double-mutant (S707A/S711A) LSD1 (which is unable to undergo effective GSK3β-mediated phosphorylation) were significantly decreased after exposure of cancer cells to cycloheximide for 2 h (Fig. 3a). As ubiquitinated proteins are efficiently degraded in the proteasome, we had to add a proteasome inhibitor MG132 to show the differential ubiquitinated levels. Ubiquitinated levels of the double-mutant (S707A/S711A) LSD1 were higher than that of the wild-type LSD1 (Fig. 3b). The protein amount of LSD1 was decreased in GSK3β-knocked down cancer cells, but such decrease could be inhibited by treatment with MG132 (Fig. 3c). When cells were exposed to the specific GSK3β inhibitor LY2090314, protein levels of LSD1 were reduced in a dose-dependent manner. In addition, histone H3 lysine 4 methylation (whose levels are reduced by LSD1 activity) were found to be increased (Fig. 3d). Finally, the demethylation capacity of double-mutant (S707A/S711A) LSD1 was absent when overexpressed in cancer cells (Fig. 3e). Taken together, these data suggest that GSK3β-mediated LSD1 phosphorylation stabilizes LSD1 protein.

### The STIP1–HSP90 complex is required for LSD1 protein stability

RNA silencing and the peptide 520^5^ were used to shed more light on the role played by the STIP1–HSP90 complex in LSD1 protein stability. Although LSD1 protein levels were decreased in STIP1-silenced cancer cells, the proteasome inhibitor MG132 restored its normal expression (Fig. 3f). For further confirmation, the peptide 520 was used to disrupt the interaction between STIP1 and HSP90^5^. The results indicated that exposure of cancer cells to the peptide 520 reduced LSD1 expression (Fig. 3g). Taken together, these data suggest that the STIP1–HSP90 complex is required for LSD1 protein stability.

### GSK3β-mediated LSD1 phosphorylation drives its subcellular localization

**Fig. 4:**
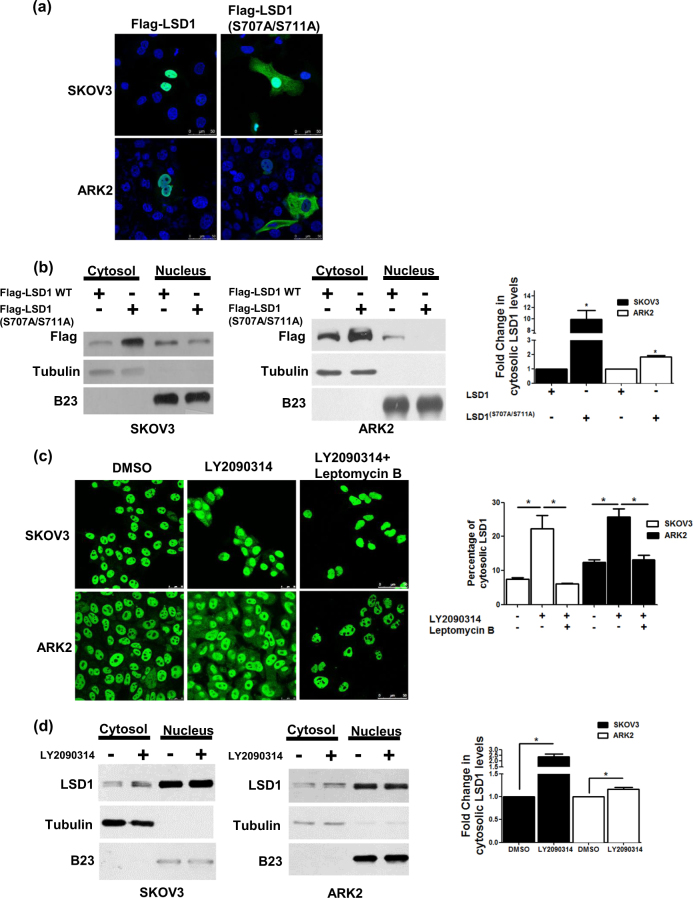
Phosphorylation of LSD1 alters its subcellular localization. **a** The subcellular localization of Flag-wild-type LSD1 or Flag-double-mutant (S707A/S711A) LSD1 (green) was investigated with confocal microscopy. **b** Nuclear and cytoplasmic fractions obtained from cancer cells (SKOV3 and ARK2) overexpressing Flag-wild-type LSD1 or Flag-double-mutant (S707A/S711A) LSD1 were analyzed with Western blot using anti-Flag, anti-tubulin (cytoplasmic marker), and anti-B23 (nuclear marker) antibodies, respectively. **c** Confocal microscopy image indicated the intracellular localization of endogenous LSD1 (green) in cells treated with DMSO or LY2090314 (10 µM) for 24 h or LY2090314 (10 µM) with leptomycin B (10 nM) for 24 h. Cytosolic LSD1 were quantified with MetaMorph software. **d** SKOV3 and ARK2 cells were treated DMSO or LY2090314 (10 µM) for 24 h. Protein levels of the nuclear or cytoplasmic fractions of LSD1, tubulin, and B23 were determined with Western blot. Results shown were obtained from three independent experiments and presented as mean  ± standard error (SE). *P < 0.05, Student’s *t*-test

Wild-type Flag–LSD1 was found to be located in the nucleus. In contrast, approximately 10% of Flag-double-mutant (S707A/S711A) LSD1 was localized in both the cytoplasm and the nucleus (Fig. 4a). To shed more light on these findings, cell fractionation experiments were conducted. The results revealed that Flag-double-mutant (S707A/S711A) LSD1 protein levels were decreased in the nucleus, whereas they were found to be increased in the cytoplasm (Fig. 4b). The specific GSK3β inhibitor LY2090314 also caused the translocation of wild-type LSD1 from the nucleus to the cytoplasm (Fig. 4c, d). The major signal of LSD1 was locked in the nucleus by leptomycin B (right panel, Fig. 4c), and signal of LSD1 in the cytoplasm only appeared in the absence of leptomycin B (middle panel, Fig. 4c). Taken together, these findings indicate that GSK3β-mediated LSD1 phosphorylation helps retention of LSD1 in the nucleus.

### GSK3β-mediated LSD1 phosphorylation promotes cell proliferation

**Fig. 5:**
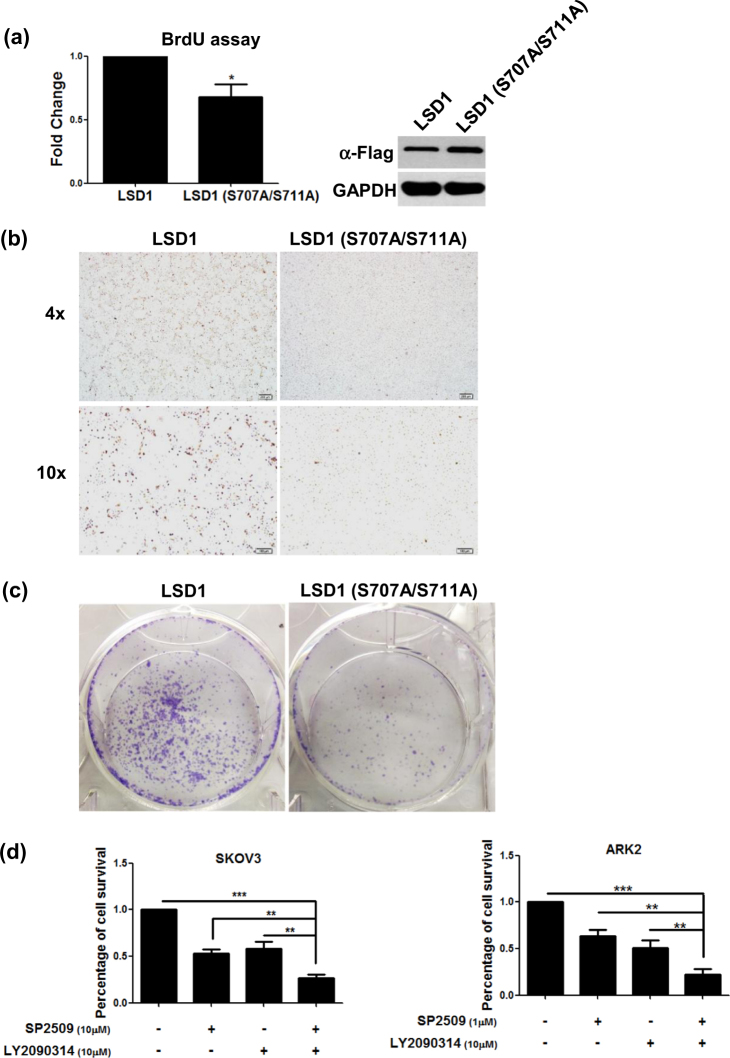

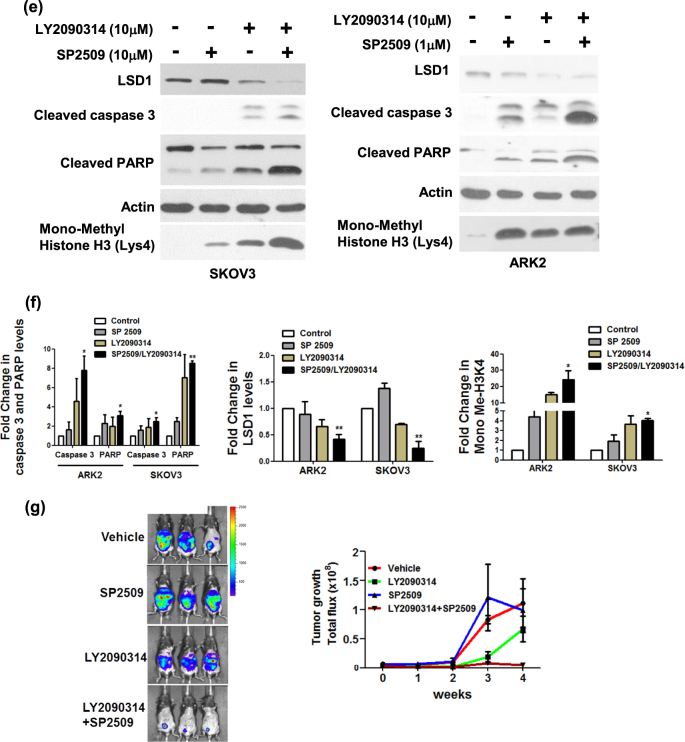
The combination of the LSD1 inhibitor SP2509 and the GSK3β inhibitor LY2090314 exerts synergistic antitumor effects. **a** BrdU proliferation assay, **b** Ki67 protein immunostaining, and **c** colony formation assay in ARK2 cells (1000 cells cultured in 1% FBS medium in 6-well plate) stably expressing Flag-wild-type LSD1 or Flag-double-mutant (S707A/S711A) LSD1. **d** SKOV3 and ARK2 cells were treated with the reported concentrations of SP2509, LY2090314, or a combination of SP2509 and LY2090314 for 72 h. Cell survival was determined with the MTT assay. **e** Exposure of SKOV3 and ARK2 cells to a combination of SP2509 and LY2090314 for 72 h increased apoptosis (as reflected by higher levels of cleaved caspase-3 and cleaved PARP quantified by Western blot). LSD1 activity was assessed by measuring protein levels of mono-methylated histone H3K4. Actin levels were used to normalize input proteins. **f** Quantified results from **e** were obtained from three independent experiments and are presented as mean ± standard error (SE). *P < 0.05, **P < 0.01, ***P < 0.001, Student’s *t*-test. **g** Mosec/Luc cells (1 × 10^6^) were intraperitoneally injected into C57BL/6 mice. One week later, mice were injected with vehicle alone, LY2090314 (10 mg/kg), SP2509 (10 mg/kg), or both twice a week. Tumor growth was measured on a weekly basis after treatment was started. Ascites were detected in mice treated with vehicle alone or SP2509. The mice were then analyzed with the IVIS Spectrum in vivo imaging system on a weekly basis. Data are expressed as means ± standard error

Cells stably expressing Flag–LSD1 or Flag-double-mutant (S707A/S711A) LSD1 were used to investigate whether GSK3β phosphorylation might be involved in LSD1-mediated cell growth. The BrdU assay and Ki67 staining were used as markers of cell proliferation. Compared with Flag–LSD1, cells stably expressing Flag-double-mutant (S707A/S711A) LSD1 showed both a decreased BrdU incorporation rate and a less prominent Ki67 staining (Fig. 5a, b). A reduced colony growth was observed in cells expressing Flag-double-mutant (S707A/S711A) LSD1 (Fig. 5c). Taken together, these data suggest that GSK3β-mediated LSD1 phosphorylation promotes cell proliferation.

### The LSD1 inhibitor SP2509 and the GSK3β inhibitor LY2090314 exert synergistic antitumor effects

To investigate whether the combination of the LSD1 inhibitor SP2509 and the GSK3β inhibitor LY2090314 could exert synergistic antitumor effects, different concentrations of the two molecules were tested. SP2509 at a 10 µM concentration caused death in 50% of SKOV3 cells, whereas a 1 µM concentration was sufficient to cause ARK2 cell death in an identical amount. A 50% cell death was observed for both cell lines when LY2090314 was used at a 10 µM concentration (Fig. 5d). Combined treatment with both SP2509 and LY2090314 in both SKOV3 and ARK2 cells exerted synergistic cytotoxic effects, ultimately increasing the cell death rate to 75% (Fig. 5d). Levels of cell death-associated proteins (including cleaved caspase-3 and cleaved poly-(ADP-ribose)-polymerase [PARP]) were also increased when the two inhibitors were used in combination (Fig. 5e, f). As expected, histone H3 lysine 4 methylation was markedly increased upon LY2090314 treatment (Fig. 5e, f). The in vivo treatment with both LY2090314 and SP2509 inhibited tumor proliferation more effectively than LY2090314 or SP2509 alone (Fig. 5g). Taken together, these results indicate that LSD1 and GSK3β inhibitors can exert synergistic antitumor effects when used in combination.

### Immunohistochemical co-expression of STIP1, LSD1, and GSK3β in human cancer tissues

**Fig. 6:**
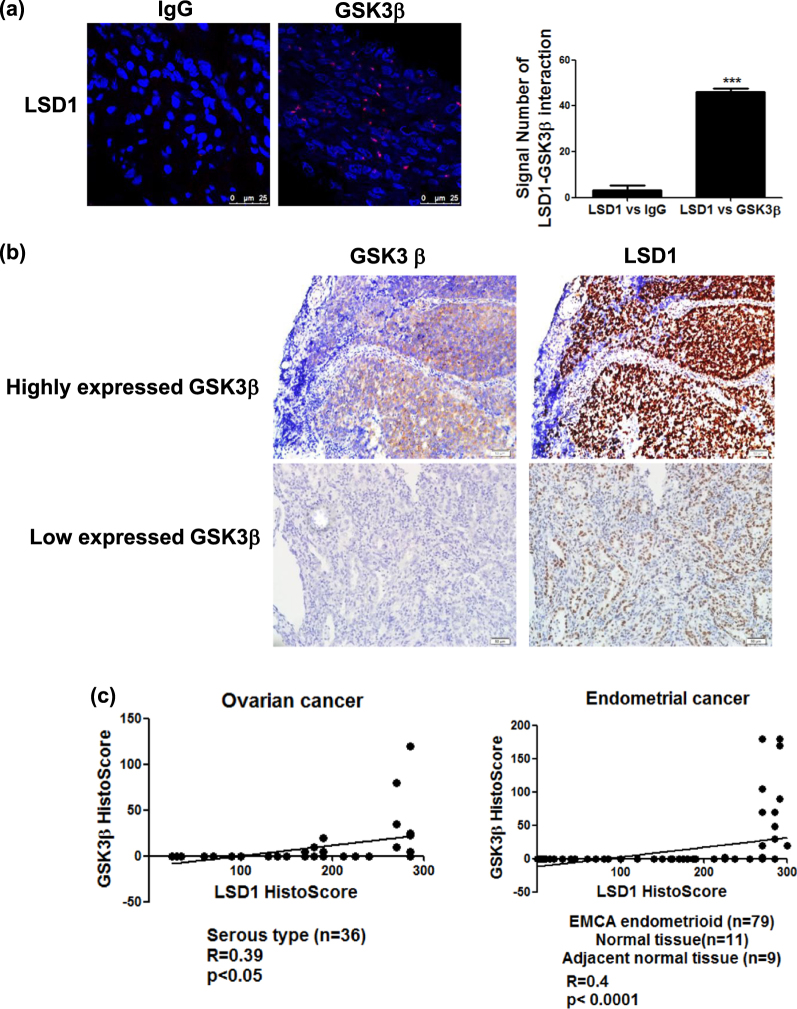

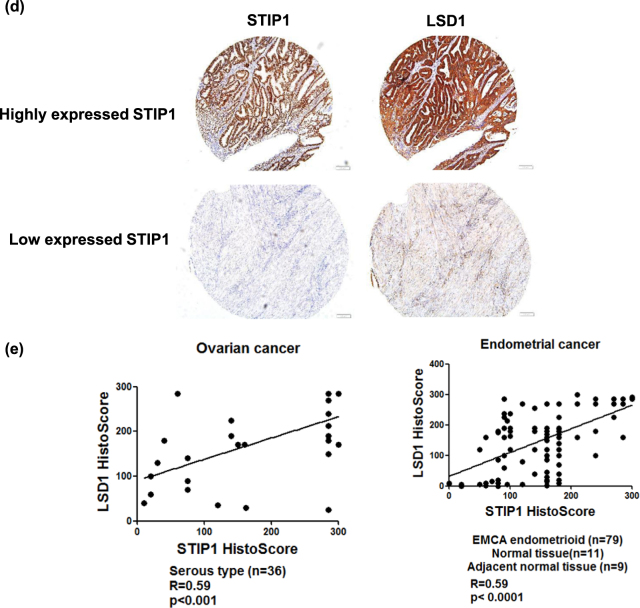
LSD1 and GSK3β are co-expressed in human cancer specimens. **a** A proximity ligation assay showed an interaction between LSD1 and GSK3β in ovarian cancer specimens. Anti-STIP1 and Anti-LSD1 antibodies were used for staining, whereas an IgG served as a negative control. The signals were counted with ImageJ (https://imagej.nih.gov/ij/) (right panel). ***P < 0.001, Student’s *t*-test. **b** The immunohistochemical expression of LSD1 and GSK3β in endometrial cancer tissues was investigated. Endometrial cancers with high and low GSK3β immunohistochemical expression are shown in the upper and lower panels, respectively. **c** A histoscore was used to analyze LSD1 and GSK3β immunostaining in ovarian cancer (*n* = 36) and endometrial tissue array (*n* = 99). **d** Immunohistochemical staining of STIP1 and LSD1 was performed in an endometrial cancer tissue array. Endometrial cancer tissues with high and low STIP1 immunohistochemical expression are shown in the upper and lower panels, respectively. **e** The correlation between STIP1 and LSD1 expression in ovarian cancer (*n* = 36) and endometrial tissue array (*n* = 99) was analyzed with a histoscore

A proximity ligation assay (PLA) was performed to identify the interactions between LSD1 and GSK3β in formalin-fixed, paraffin-embedded endometrial cancer specimens. The results revealed that the interaction between LSD1 and GSK3β mainly occurred in the nucleus (Fig. 6a). An endometrial cancer tissues with high expression of GSK3β exhibited higher levels of LSD1 (upper panels of Fig. 6b), whereas the GSK3β-lowly expressing one had lower levels of LSD1 (lower panels of Fig. 6b). Positive correlations between LSD1 and GSK3β expression were also identified in ovarian (Fig. 6c, left panel, r = 0.39, *P* < 0.05) and endometrial cancer (Fig. 6c, right panel, *r* = 0.4, *P* < 0.0001) specimens. A STIP1 highly expressing endometrial cancer tissues exhibited higher levels of LSD1 (upper panels of Fig. 6d), whereas a STIP1 lowly expressing one had lower levels of LSD1 (lower panel of Fig. 6d). The immunohistochemical expression of STIP1 and LSD1 showed positive associations in both ovarian (Fig. 6e, left panel, *r* = 0.59, *p* < 0.001) and endometrial cancer (Fig. 6e, right panel, *r* = 0.59, *p* < 0.0001) tissues. Taken together, these preliminary data suggest that the interactions between STIP1, LSD1, and GSK3β observed in vitro do also occur in vivo.

## Discussion

**Fig. 7:**
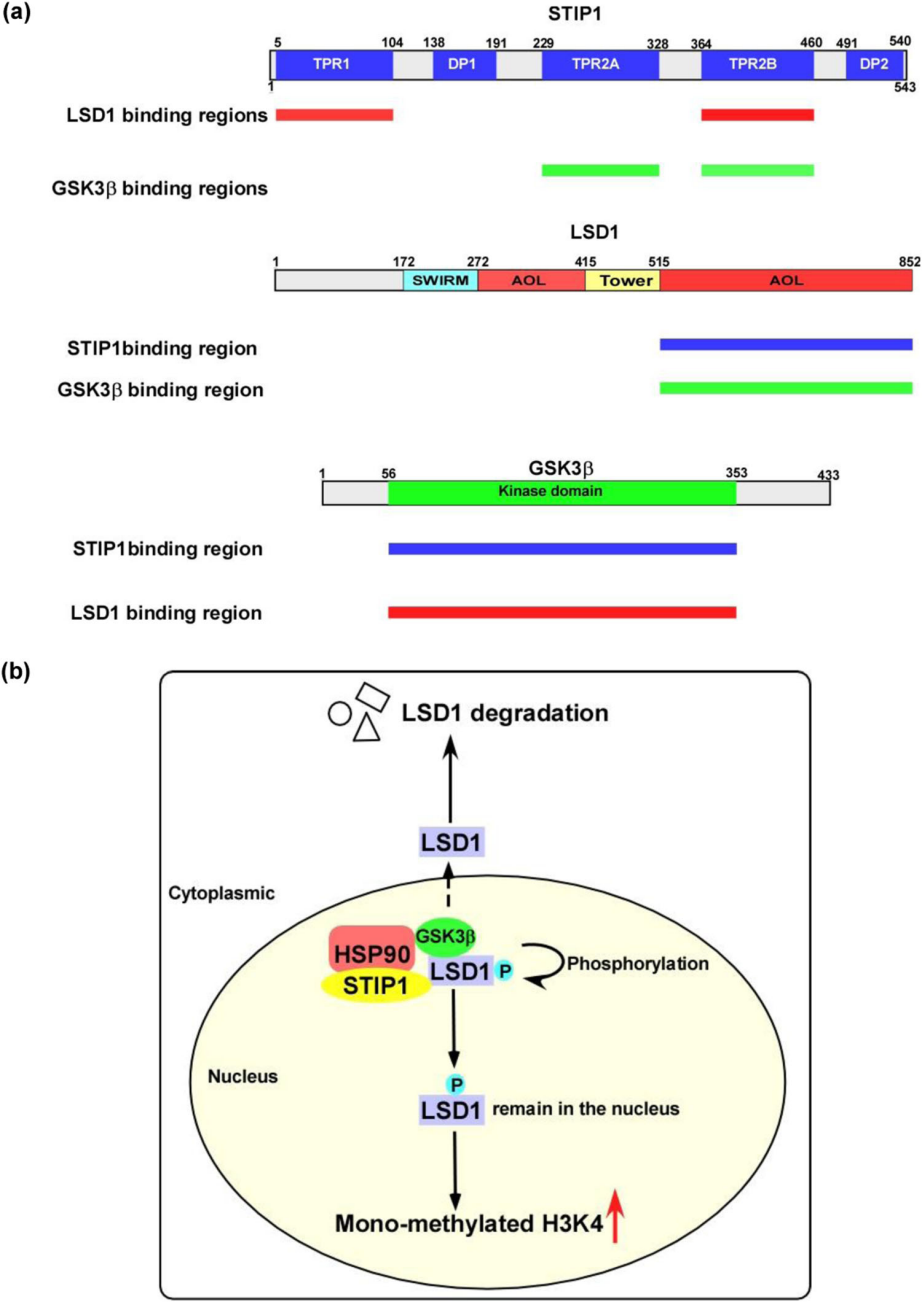
Schematic representation of the role played by the STIP1–HSP90 complex in GSK3β-mediated LSD1 phosphorylation. **a** Summary of the interactions between STIP1, LSD1, and GSK3β. **b** The STIP1–HSP90 complex acts as a scaffold promoting GSK3β-mediated LSD1 phosphorylation. When GSK3β-mediated LSD1 phosphorylation is inhibited, LSD1 translocates from the nucleus into the cytoplasm—where it undergoes degradation

Herein, we unveiled the existence of a complex molecular interplay between STIP1, LSD1, and GSK3β (Fig. 7a), that ultimately provides new mechanistic evidences on the role played by STIP1 in human tumorigenesis. First in the literature, our findings show that the STIP1–HSP90 complex acts as a scaffold to bring LSD1 and GSK3β together, thereby promoting GSK3β-mediated LSD1 phosphorylation (Fig. 7b). Specifically, experiments with systematically truncated constructs of STIP1 revealed that its TPR1 domain interacts with the C-terminal AOL domain of LSD1, whereas its TPR2A and TPR2B domains are capable of binding with the GSK3β kinase domain (Figs. 1 and 7a). Intriguingly, LSD1 and GSK3β were unable to form a complex in the absence of STIP1 (Fig. 1d–f). We also demonstrated that GSK3β-mediated LSD1 phosphorylation resulted in an increased LSD1 protein stability, associated with its nuclear expression and an effective demethylase activity. It has been previously reported that GSK3β phosphorylates and stabilizes LSD1 through the binding with the USP22 deubiquitinase^35^. Our results confirm and expand published data on the key role played by GSK3β-mediated LSD1 phosphorylation at specific sites in promoting its stability.

Upregulation of STIP1 in various cancers^7–9,11,12,14,36–38^ is recently found to be regulated by both RAS activation and p53 inhibition^39^. Nuclear HSP90 chaperone complexes are involved in transcriptional regulation in nucleus^40^. HSP90 and its co-chaperones can form a complex with transcription factors and control gene expression through chromatin remodeling, histone modification or RNA polymerase II regulation^40^. However, it is unclear how HSP90 be translocated into the nucleus in the absence of nucleus localization sequence (NLS)^41,42^. HSP90 may interact with other proteins along with NLS that translocate into the nucleus^41,42^. In contrast, the mechanisms of STIP1 translocation are documented. Cytoplasmic STIP1 is promoted by Cdc2 kinase phosphorylation, whereas the translocation of STIP1 is freely interacting with HSP90 in cytoplasm and controlled by Casein kinase II (CK2) phosphorylation^43,44^. STIP1 can also interact with Protein Inhibitor of Activated STAT1 (PIAS1) for nuclear retention^45^. As far as the key role of STIP1 as a scaffold protein is concerned, our data suggest that knockdown of endogenous STIP1 decreased both the LSD1/HSP90 interaction (Fig. 1d) and LSD1 protein levels (Fig. 3f). Interestingly, STIP1 gene silencing via siRNA or the use of a specific peptide disrupting the STIP1–HSP90 complex also blocked the interaction between LSD1 and GSK3β (Figs. 1e, f). In light of these findings, we hypothesize that HSP90–GSK3β complex translocates into the nucleus through NLS in GSK3β and that STIP1 may translocate by CK II phosphorylation, interact with LSD1 in the nucleus and subsequently promote its transfer to the HSP90–GSK3β complex, ultimately resulting in both appropriate LSD1 folding and subsequent phosphorylation.

Besides promoting protein stability, our results revealed that GSK3β-mediated LSD1 phosphorylation was capable of regulating LSD1 cellular localization and demethylating functions. In particular, LSD1 was translocated from the nucleus to the cytoplasm in presence of a GSK3β inhibitor (Fig. 4c, d). Similar findings were observed in cancer cells overexpressing double-mutant (S707A/S711A) LSD1 (Fig. 4a, b). Notably, demethylase activity—which governs histone H3 lysine 4 methylation levels—was decreased by the exposure to a GSK3β inhibitor even at low concentrations (Fig. 3d). Double-mutant (S707A/S711A) LSD1 similarly increased histone H3 lysine 4 methylation levels. We speculate that GSK3β-mediated phosphorylation maintains the nuclear localization of LSD1 and regulates its demethylase activity. In support of our hypothesis, the pharmacological inhibition of GSK3β promoted the following events: (1) translocation of LSD1 from the nucleus to the cytoplasm, (2) loss of its demethylase activity, and (3) its ultimate degradation.

The molecular chaperone HSP90 utilizes ATP as an energy source for recruiting a variety of client proteins involved in different signaling pathways^32^. Specific HSP90 inhibitors promote the degradation of HSP90 target proteins via the ubiquitin-proteasome pathway. We have previously shown that HSP90 inhibitors are capable of regulating JAK2 kinase stability^5^. Here, we demonstrate that HSP90 may interact with GSK3β to maintain its stability. Published data also suggest that GSK3β is degraded and its kinase activity is abrogated by HSP90 inhibitors^33,46^. Interestingly, an in vitro study revealed that GSK3β can phosphorylate HSP90 and stimulate its binding to STIP1 in cells characterized by a high proliferative potential^47^. Taken together, there is ample evidence that GSK3β is able to interact and phosphorylate HSP90. Upon phosphorylation, HSP90 acquires the capacity of binding to the STIP1–LSD1 complex, ultimately promoting the translocation of LSD1 to GSK3β.

Interest in the antiproliferative potential of LSD1 and GSK3β inhibitors has recently gained momentum. SP2509 is a highly potent polyamine-based, reversible, and specific inhibitor of LSD1 that acts as a non-MAO-A and non-MAO-B inactivator^48^. SP2509 has been shown to inhibit tumor cell proliferation in solid malignancies (e.g., Ewing sarcoma, colorectal, breast, and endometrial cancers)^48,49^, as well as in acute myeloid leukemia^50^. In general, LSD1 inhibitors block its demethylase activity and activate target genes by restoring histone H3 lysine 4 methylation^51^. As far as GSK3β inhibitors are concerned, LY2090314 is currently under investigation in a phase II trial of refractory/recurrent acute myeloid leukemia (clinicaltrials.gov identifier NCT01214603). Interestingly, we show here that LSD1 and GSK3β inhibitors used in combination were able to increase both cell death rates and levels of cell death-related proteins (i.e., cleaved caspase-3 and cleaved PARP; Fig. 5d, e) compared with their use as monotherapy. This observation—coupled with the positive correlation between LSD1 and GSK3β immunohistochemical expression in human cancer specimens—may prompt further preclinical studies on the combined used of LSD1 and GSK3β inhibitors.

In summary, our current data provide mechanistic insights into the role of STIP1 in human tumorigenesis by showing that it serves as a scaffold for GSK3β-mediated LSD1 phosphorylation. The combination of LSD1 and GSK3β inhibitors may exert synergistic antitumor effects and deserves further scrutiny in preclinical studies.

## Materials and methods

### Culture, treatment, and transfection of cell lines

Human ovarian cancer cells SKOV3, ES2, and 293 were obtained from the American Type Culture Collection (Manassas, VA, USA). Human ovarian cancer cells Kuramochi cells were purchased from the Japanese Collection of Research Bioresources Cell Bank (Osaka, Japan). The human endometrial cancer cell line ARK2 was kindly provided by Dr. Alessandro D. Santin (Yale School of Medicine, New Haven, CT, USA). Mouse ovarian surface epithelial cancer cell line (Mosec/Luc) was a gift from Dr. Chi-Long Chang (Institute of Biomedical Sciences, Mackay Medical College, Taiwan). SKOV3 and 293 cells were cultured in DMEM/F12^5,52^, whereas ARK2, Kuramochi and Mosec/Luc cells were maintained in RPMI medium. In pharmacological experiments, cells were pre-treated with the proteasome inhibitor MG132 (10 µM concentration; Sigma, St. Louis, MO, USA), the protein synthesis inhibitor cycloheximide (25 µg/ml concentration, Sigma), the LSD1 inhibitor SP2509 (working concentration as indicated in figures; Selleck Chemicals, Houston, TX, USA), the GSK3β inhibitor LY2090314 (working concentration as indicated in figures; Selleck Chemicals), leptomycin B (10 nM in working concentration, Cell signaling, Danvers, MA), Antp-TPR^34^ (20 µM working concentration; sequence: RQIKIWFQNRRMKWKKKAYARIGNSYFK; GeneDireX, Las Vegas City, NE, USA), and peptide 520^5^ (10 µM working concentration; sequence: (D-Arginine)_8_-EHLKNPVIAQKIQKLMDVGLIAIR; Kelowna International Scientific, Taipei, Taiwan). To generate stably expressed wild-type LSD1 or LSD1 S707A/S711A cells, ARK2 cells were transfected with pLAS5w.PeGFP-I2-Puro-LSD1 or pLAS5w.PeGFP-I2-Puro-LSD1 S707A/S711A and treated with 5 μg/ml puromycin (Invitrogen, Carlsbad, CA, USA) at 48 h after transfection. One month later, the individual clones were selected and assayed for the LSD1 expression levels. siRNA and DNA transfection in ARK2 and SKOV3 were performed as previously described^5,53^.

### DNA construction

Full-length and truncated LSD1 constructs were generated as described previously^54^. HA- GSK3β was a gift from Dr. Chi-Neu Tsai (Chang-Gung University, Taoyuan, Taiwan).The pNTAP-GSK3β deletion constructs were amplified and cloned into pNATP vector using an In-Fusion HD cloning kit (Clontech, Mountain View, CA, USA). The double-mutant (S707A/S711A) LSD1 and HA-GSK3β S9A were generated by overlapping PCR with a Q5 Site-Directed Mutagenesis Kit (New England Biolabs) according to the manufacturer’s protocols. To construct the expression vector for the stably expression in cancer cells, the PCR products were amplified from Flag–LSD1 and Flag–LSD1 S707A/S711A and cloned into pLAS5w.PeGFP-I2-Puro (RNAi core laboratory, Sinica, Taiwan) with In-Fusion HD cloning kit (Clontech, Mountain View, CA, USA) according to the manufacturer’s protocols. The primers used in DNA constructs are shown in Supplementary Table 1. The procedure was confirmed by DNA sequencing.

### Western blot analysis

Western blot were performed as described previously^5,54^. All of the antibodies used for the experiments were obtained from commercial sources, as follows: LSD1, mono-methylated histone 3 lysine 4, total histone 3, and GSK3β were from Cell Signaling Technology (Danvers, MA, USA); calmodulin binding peptide tag (CBP) was from Millipore (Billerica, MA, USA); halo tag was from Promega (Madison, WI, USA); actin and STIP1 were from Santa Cruz Biotechnology (Santa Cruz, CA, USA). All corresponding horseradish peroxidase-conjugated antibodies were obtained from Santa Cruz Biotechnology. Enhanced chemiluminescence reagents were from Millipore. The signal intensity of autoradiograms was quantified using the ImageJ software (https://imagej.nih.gov/ij/) after normalization with the corresponding actin signal intensity.

### Cell fractionation

After trypsinization and washing with cold PBS, cells were re-suspended in hypotonic buffer (20 mM Tris-HCl, pH 7.4, 10 mM NaCl, 3 mM MgCl_2_, 10% NP-40) and the supernatant containing the cytoplasm fraction was collected by centrifugation. The leftover nuclear pellets were lysed in cell extraction buffer (100 mM Tris-HCl, pH 7.4, 2 mM Na_3_VO_4_, 100 mM NaCl, 1% triton X-100, 1 mM EDTA, 10% glycerol, 1 mM EGTA, 0.1% SDS, 1 mM NaF, 0.5% deoxycholate, and 20 mM Na_4_P_2_O_7_).

### Immunoprecipitation

Cell lysates were prepared with Cell lysis buffer (20 mM Tris-Cl pH 7.4, 25 mM NaCl and 0.1% NP-40) with the cocktails of phosphatase inhibitors (50 μg/ml Sodium Fluoride, 0.2 mg/ml Sodium Orthovanadate, 0.3 mg/ml Sodium Molybdate, 1.2 mg/ml Sodium Tartrate, 0.15 mg/ml Imidazole, 1 μg/ml Cantharidin, 7.5 μg/ml (−)-*p*-Bromotetramisole, 5 ng/ml Microcystin LR; Bionovas biotechnology, Toronto, Canada) and proteinase inhibitors (25 μg/ml AEBSF, 2 μg/ml Aprotinin, 0.3 μg/ml Bestatin, 0.4 μg/ml E-64, 1 μg/ml Leupeptin, 1 μg/ml Pepstatin A, 10 μg/ml Benzamidine HCl, 1.2 μg/ml Phosphoramidon, 1 mg/ml 1,10-phenanthroline; Bionovas biotechnology, Toronto, Canada) and performed as previously described^5,54^. Western blot analysis was performed using the antibody for calmodulin binding peptide (CBP; Millipore) and halo tag (Promega).

### Proximity ligation assay

Proximity ligation assay was performed with Duolink In Situ Starter Kit (Sigma) according to manufacture’s protocol as described previously^5^. The slides were stained with a combination of LSD1 (Cell Signaling Technology), GSK3β (Santa Cruz Biotechnology) antibodies, as well as an IgG control antibody (Sigma) The slides were then examined using a Leica TCS SP2 confocal laser scanning microscope (Leica Microsystems GmbH, Wetzlar, Germany) and the numbers of interaction signal was quantified using the ImageJ software

### Immunofluorescence microscopy

Briefly, After DNA transfection, cells were fixed with 2% paraformaldehyde at 4 °C for 30 min and then incubated for 1 h at room temperature in blocking buffer (Thermo Fisher Scientific, MA, USA)^5^. Flag and LSD1 staining was performed by incubating cells with mouse monoclonal antibodies raised against Flag (M2, Sigma; 1:100 dilution) and LSD1 (Cell Signaling Technology; 1:100 dilution). After incubation with anti-Alexa Fluor-488 (Invitrogen; 1:100 dilution), slides were mounted with the mounting medium (Sigma Aldrich) and analyzed using a Leica TCS SP2 laser scanning confocal system (Leica Microsystems GmbH). The average integration of fluorescence was quantified with MetaMorph image analysis software (Molecular Devices, LLC., Sunnyvale, CA).

### Ki67 immunostaining and BrdU assay

For BrdU assay, ARK2 cells were treated with BrdU for 2 h after transfecting with wild-type LSD1 or LSD1 S707A/S711A for 48 h. DNA synthesis activity was measured with BrdU ELISA kit (Roche applied science). For Ki67 staining, after tranfecting with wild-type LSD1 or LSD1 (S707A/S711A) in ARK2 cells for 48 h^8^, Ki67 was stained with an anti-Ki67 antibody (Cell Signaling Technology).

### Immunohistochemistry

This immunohistochemical study complied with the tenets of the Helsinki declaration and was approved by our local Institutional Review Board (No.101-4771B). Formalin-fixed, paraffin-embedded ovarian cancer tissues and endometrial cancer tissue array (UT1002, US Biomax. Inc, Rockville, MD, USA) were sectioned at 4 μm thickness and deparaffinized with xylene. Sections were dehydrated through a series of graded ethanol baths and stained with antibodies raised against human LSD1 (Cell Signaling Technology) and GSK3β (Cell Signaling Technology) in an automated immunohistochemical stainer (Leica Bond Polymer Refine Detection Kit; Buffalo Grove, IL, USA) according to the manufacturer’s protocol. Hematoxylin was used for counterstaining. In line with previous methodology^53^, a semiquantitative assessment of immunohistochemical staining was performed using a histoscore calculated by multiplying the percentage of tumor cells (from 0 to 100%) by their immunointensity (from 0 to 3).

### In vivo animal model

Briefly, after inoculating Mosec/luc cells to C57BL/6 mouse 1 week later^52^, mice were injected intraperitoneally with designated regimens: vehicle control (20% Water: 20% DMSO: 60% cremophor), LY2090314 (10 mg/kg), SP2509 (10 mg/kg), and LY2090314 plus SP2509 (10 mg/kg each) twice a week. Tumor growth was monitored by luciferase activity detected with the Xenogen IVIS Spectrum In Vivo Imaging System (Xenogen Corp., Alameda, CA, USA). Light outputs were quantified using the LivingImage software (Xenogen Corp.).

## Electronic supplementary material

Supplementary informaiton(DOCX 18 kb)
