# Supplementary material for: Stress-induced phosphoprotein 1 acts as a scaffold protein for glycogen synthase kinase-3 beta-mediated phosphorylation of lysine-specific demethylase 1
Source: Oncogenesis. 2018 Mar 29;7(3):31. doi: 10.1038/s41389-018-0040-z (PMC5874249; doi:10.1038/s41389-018-0040-z)
Supplement: Supplementary file 1 — Supplementary informaiton(DOCX 18 kb) [file 41389_2018_40_MOESM1_ESM.docx]

| Primer | Sequence |
| --- | --- |
| GSK3β-Forward | CTGCCCGGGCGGATCCATGTCGGGGCGACCG |
| GSK3β-Reverse | CGGTATCGATAAGCTTGGTGGAGTTGGAAGCTGATG |
| GSK3β-Reverse(1~56) | CGGTATCGATAAGCTTGGTGGAGTTGGAAGCTGATG |
| GSK3β-Reverse(1~353) | CGGTATCGATAAGCTTAAAAAATGAATGTGCACAAGCT |
| GSK3β-Forward (56~433) | CTGCCCGGGCGGATCCACAGACACTAAAGTGATTGGAAATG |
| GSK3β-Forward (353~433) | CTGCCCGGGCGGATCCGATGAATTACGGGACCCAAAT |
| GSK3β-Forward (56~353) | CTGCCCGGGCGGATCCACAGACACTAAAGTGATTGGAAATG |
| GSK3β-Reverse (56~353) | CGGTATCGATAAGCTTAAAAAATGAATGTGCACAAGCT |
| LSD1 S707A/S711A forward | TGCCGCCAGGGGTGAGCTCTTCCTC |
| LSD1 S707A/S711A Reverse | GTCGTAGCGCCAACATGCCCGAACAA |
| GSK3β S9A Forward | GAGAACCACCGCCTTTGCGGA |
| GSK3β S9A Reverse | GGTCGCCCCGACATGAAT |
| Lenti-LSD1 Forward | TCGAACCTTAGGGATATCATGGACTACAAAGACGATGAC |
| Lenti-LSD1 Reverse | TCAAGATCTAGAATTCGATATCCATGCTTGGGGACTGCTG |

**Supplementary Table 1: Primers for DNA constructs**
